# nArgBP2 together with GKAP and SHANK3 forms a dynamic layered structure

**DOI:** 10.3389/fncel.2024.1354900

**Published:** 2024-02-19

**Authors:** Sang-Eun Lee, Sunghoe Chang

**Affiliations:** ^1^Department of Physiology and Biomedical Sciences, Seoul National University College of Medicine, Seoul, South Korea; ^2^Neuroscience Research Institute, Seoul National University College of Medicine, Seoul, South Korea

**Keywords:** nArgBP2, shank, GKAP, dendritic spines, phase-separation

## Abstract

nArgBP2, a protein whose disruption is implicated in intellectual disability, concentrates in excitatory spine-synapses. By forming a triad with GKAP and SHANK, it regulates spine structural rearrangement. We here find that GKAP and SHANK3 concentrate close to the synaptic contact, whereas nArgBP2 concentrates more centrally in the spine. The three proteins collaboratively form biomolecular condensates in living fibroblasts, exhibiting distinctive layered localizations. nArgBP2 concentrates in the inner phase, SHANK3 in the outer phase, and GKAP partially in both. Upon co-expression of GKAP and nArgBP2, they evenly distribute within condensates, with a notable peripheral localization of SHANK3 persisting when co-expressed with either GKAP or nArgBP2. Co-expression of SHANK3 and GKAP with CaMKIIα results in phase-in-phase condensates, with CaMKIIα at the central locus and SHANK3 and GKAP exhibiting peripheral localization. Additional co-expression of nArgBP2 maintains the layered organizational structure within condensates. Subsequent CaMKIIα activation disperses a majority of the condensates, with an even distribution of all proteins within the extant deformed condensates. Our findings suggest that protein segregation via phase separation may contribute to establishing layered organization in dendritic spines.

## Introduction

nArgBP2 is a neural isoform of ArgBP2 (Arg binding protein 2, also known as SORBS2) that possesses a sorbin homology domain, three Src homology 3 domains, and a zinc-finger motif. It is highly expressed in brain regions linked to bipolar disorder and intellectual disability (Zhang et al., 2016). nArgBP2 interacts with diverse proteins like α2-spectrin, synaptojanin1/2, vinculin, Abl, Cbl, dynamin1, WAVE1/2, PIR121, and Nap1, modulating actin cytoskeleton dynamics and balancing adhesion with motility through various signaling pathways (Cestra et al., 2005).

nArgBP2 primarily localizes in dendritic spines of excitatory spiny pyramidal neurons, where it interacts with guanylate kinase-associated protein (GKAP, also known as SAPAP, SAP90/PSD-95-associated protein) and indirectly with SHANK through GKAP, thus forming a structural and functional scaffolding network within dendritic spines (Kawabe et al., 1999; Lee et al., 2016, 2018). Our previous work demonstrated that nArgBP2 ablation in developing neurons markedly alters spine formation and selectively suppresses excitatory spine-synapse development, leading to an excitatory-inhibitory synaptic imbalance (Lee et al., 2016).

The multivalency and conformational flexibility of post-synaptic scaffold proteins, driven by extensive protein-protein interactions, suggest their potential role in forming biological condensates through liquid-liquid phase separation (LLPS). SynGAP, a representative post-synaptic scaffolding protein, exhibits LLPS behavior, forming condensates *in vitro* that could act as a platform for recruiting PSD-95 and receptors to generate excitatory synapses (Zeng et al., 2016a). Recent studies also demonstrate LLPS in additional post-synaptic proteins like GKAP, SHANK, Homer, and GluN2B (Zeng et al., 2018). We recently also found that nArgBP2 undergoes LLPS both *in vitro* and in mature neurons, thereby regulating its function in the spatiotemporal control of structural synaptic plasticity (Cho et al., 2023).

Recent advances in super-resolution microscopy together with electron microscopy have provided new insights into the lateral organization within the post-synaptic density (PSD) (Petersen et al., 2003; Chen et al., 2008, 2018; Tao-Cheng et al., 2015; Dosemeci et al., 2016). Instead of being uniformly distributed, PSD proteins were found to be clustered into rather distinct subsynaptic domains (Zeng et al., 2018). The outer face of the PSD is enriched with neurotransmitter receptors and trans-synaptic adhesion molecules embedded within the plasma membrane. Positioned beneath these receptors, within 30–40 nm from the post-synaptic membrane, is a densely populated matrix of proteins including scaffold proteins (PSD-95, PSD-93, SAP102, and SAP97), actin-binding proteins, and downstream signaling molecules (DeGiorgis et al., 2006). GKAP, CRIPT, and IRSp53 are located in the intermediate zone of the PSD (Funke et al., 2005; Gerrow et al., 2006), while the SHANK family occupies the cytoplasmic margin of the PSD (∼40 to ∼100 nm from the post-synaptic membrane) (Naisbitt et al., 1999). The distinct layered organization and the subsynaptic domains within the PSD suggest the intriguing possibility that these highly organized sub-segregations may comprise protein condensates driven by LLPS (Zeng et al., 2018; Zeng et al., 2019a,b; Chen et al., 2020).

Diverse psychiatric disorders like schizophrenia (SCZ), bipolar disorder (BD), obsessive compulsive disorder (OCD), and autism spectrum disorder (ASD) may share a common underlying mechanism, as suggested by a large-scale study (Cross-Disorder Group of the Psychiatric Genomics et al., 2013; Uher and Zwicker, 2017). This link extends beyond clinical observations, with research revealing overlapping risk genes across these disorders (Doherty and Owen, 2014; O’Connell et al., 2018). For instance, SAPAP2 shows involvement in all three conditions (Welch et al., 2007; Pinto et al., 2010; Li et al., 2014). Similarly, SHANK3 is linked to autism (Durand et al., 2007), BD-like maniac behaviors (Han et al., 2013), and SCZ (De Sena Cortabitarte et al., 2017). Additionally, nArgBP2 deletions are associated with both intellectual disability and BD (Feng, 2010; Zhang et al., 2016). Based on these findings, we proposed a “core scaffolding triad” of these genes, suggesting their dysfunction might impact spine structure and synaptic integrity (Lee et al., 2018). However, the subcellular organization of these proteins within spines remains unexplored.

In this study, we showed that the majority of nArgBP2 is located in the lower stratum of dendritic spines, ∼ 500 nm from the synaptic membrane, positioned at a distance from SHANK3 and GKAP. We further found that these proteins form biomolecular condensates in living fibroblasts. Remarkedly, these proteins autonomously exhibit layered organizations within the condensates, mirroring established patterns within neurons and highlighting a conserved structural arrangement across cellular environments. The LLPS data we report here may explain our finding that these three proteins display a layered post-synaptic organization within dendritic spines. We suggest that the strategic positioning of nArgBP2 in both the upper and lower strata of spines is consistent with a dual function as a scaffold linker and an actin-regulating protein within dendritic spines.

## Materials and methods

Animal experiments were approved by the Institute of Animal Care and Use Committee (IACUC, Approval ID number: SNU-100930-5) of Seoul National University, Korea. All experiments were carried out in accordance with approved guidelines and regulations.

### DNA constructs and antibodies

EGFP – nArgBP2_959–1196_ was generated as previously described (Cho et al., 2023). Plasmids encoding HA-SHANK3 and GKAP-Myc were kind gifts from Prof. Jae-Yong Park (Korea University, Seoul, South Korea) and Eunjoon Kim (KAIST, Daejeon, South Korea), respectively. CaMKIIα-SBFP2 was constructed by subcloning CaMKIIα from CaMKIIα-Venus (Addgene) by PCR in SBFP2-N1 vector. RNA interference-mediated nArgBP2 knockdown was carried out by expressing small hairpin RNA shRNA) duplexes in the pSiren-U6-mRFP vector (Clontech, Palo Alto, CA) and shRNA-resistant form of EGFP-nArgBP2 (henceforth, refer to as just EGFP-nArgBP2) was used as previously described (Cho et al., 2023). Anti-HA frankenbody (15F11-HA scFv-mCherry, Plasmid #129591; Addgene) were used to label HA-SHANK3 for FRAP experiment. Primary antibodies used for immunocytochemistry are anti-rat HA (Roche, 11867431001) and anti-mouse c-Myc (sc-40; SantaCruz). Alexa Fluor™-647/405 labeled secondary antibodies were purchased from Thermo Fisher Scientific (Waltham, MA).

### Primary neuron culture and transfection

Primary rat hippocampal neurons derived from embryonic day 18 Sprague Dawley fetal rats of either sex were prepared as described previously (Lee et al., 2016). Briefly, hippocampi were dissected, dissociated with papain (Worthington Biochemical Corporation, Lakewood, NJ), and resuspended in minimal Eagle’s medium (MEM, Invitrogen) supplemented with 0.6% glucose, 1 mM pyruvate, 2 mM L-glutamine, and 10% fetal bovine serum (Hyclone, South Logan, UT), and plated on poly-D-lysine-coated glass coverslips in 60 mm Petri dishes. Four hours after plating, the medium was replaced with neurobasal medium (Invitrogen, Carlsbad, CA) supplemented with 2% B-27 (Invitrogen), 0.5 mM L-glutamine. Neurons were transfected by a modified calcium-phosphate method as previously described (Lee et al., 2016). Briefly, 6 μg of DNA and 9.3 μl of 2 M CaCl_2_ were mixed in distilled water to a total volume of 75 μl and the same volume of 2x BBS [50 mM BES, 280 mM NaCl, and 1.5 mM Na_2_HPO_4_ (pH 7.1)] was added. The cell culture medium was completely replaced by transfection medium (MEM; 1 mM sodium pyruvate, 0.6% glucose, 10 mM HEPES, 1 mM Kynurenic acid, and 10 mM MgCl_2_, pH 7.71), and the DNA mixture was added to the cells and incubated in a 5% CO_2_ incubator for 60 min. Cells were washed with a washing medium (pH 7.30) and then returned to the original culture medium. Neurons were transfected at days *in vitro* (DIV) 8–9 and analyzed at DIV 19–21.

### Cell culture and transfection

COS7 cells were cultured at 37 C in 5% CO_2_ in DMEM (Invitrogen) supplemented with 10% fetal bovine serum, and transfected with constructs using PEI (MW 4000) (Polysciences, Warrington, PA) at a ratio of 1:4 [total DNA (μg) to PEI (μL)].

### Immunocytochemistry (ICC)

COS7 cells and primary neurons were fixed for 15 min at room temperature (RT) in 4% (w/v) PFA, 4% (w/v) sucrose in PBS, pH 7.4 and subsequently permeabilized with 0.25% Triton X-100 in PBS for 3 min at RT. The cells were then blocked for 1 h at RT in 10% (w/v) Bovine serum albumin (BSA). Cells were incubated at 4°C overnight in primary antibodies (1/1000) after which the cells were washed in PBS and incubated with secondary antibodies (1/1,000) for 1 h at RT. For 1,6-hexanediol (1,6-HD) treatment, COS7 cells in Tyrode’s solution (119 mM NaCl, 2.5 mM KCl, 2 mM CaCl_2_, 2 mM MgCl_2_, 25 mM HEPES, pH 7.4 and 30 mM glucose) were imaged at 5 s intervals and exposed to 3% 1,6-hexanediol (Sigma). For ionomycin treatment, ionomycin (Sigma) was added to a working concentration of 10 μM in Tyrode’s solution and treated on the cells for 1 min before fixation.

### Super-resolution imaging and analysis

Super-resolution synapse images were acquired on a Zeiss LSM 980 microscope with Airyscan detector using a 63x, 1.4 NA oil immersion Plan-Apochromat objective (Carl Zeiss, Oberkochen, Germany). The z-step size was 130 nm, with 12 steps per Z-stack. Scan speed was 5, line averaging 2, gain 800, and digital gain 1. A laser power of 5, 3, 2, and 1% was used for the 405 nm, 488 nm, 561 nm and 647 lasers, respectively. The Airyscan detector was aligned before imaging. Imaris software (Bitplane AG, Zurich, Switzerland) was used for SHANK, nArgBP2, and GKAP puncta detection. Channel brightness was adjusted to maximize the visualization of the immunoreactive region and the surface function was then used to generate volumes representing SHANK, nArgBP2 and GKAP. A horizontal line scan across the center of the droplet was generated for the line scan. An incorporation index for *in vitro* scaffolding experiments (I_*inc*_, Figure 2D) was calculated as the ratio of average intensity in a circle region of interest (ROI) at the center of a condensate, divided by the average intensity in the outer five pixels of the condensate.

### FRAP assays

Experiments were performed using the stimulus-setting menu in the Nikon A1 to control sequential image acquisition using a 60X oil-immersion lens (1.40 N.A.) equipped with a Nikon A1 confocal microscope (Nikon) to accomplish photobleaching of a circular or cylindrical ROI by laser pulse emission. ROIs containing single droplets of COS7 cells were imaged every 5 s. After 3 images had been acquired, the droplet was photobleached for 1 s with a 488/561 nm laser (100%) and fluorescence recovery was imaged at 5 s intervals at 37°C. Average intensity values of ROI and total image fluorescence were obtained from each FRAP image using Nikon imaging software (NIS-elements). ROI values over time were plotted. Fluorescence intensities in the bleached ROIs were normalized to initial values.

### Statistics

The normality of data was examined with the Kolmogorov-Smirnov normality test. When the normality of data could not be assumed, the Friedman test was used for the non-parametric comparison of multiple groups. Prism 10 (GraphPad Software, San Diego, CA) was used for statistical analysis.

## Results

### nArgBP2, GKAP, and SHANK are spatially situated in distinct regions of the dendritic spines

nArgBP2 interacts with GKAP, and GKAP interacts with SHANK (Kawabe et al., 1999; Zeng et al., 2016b; Lee et al., 2017). Although nArgBP2 and SHANK do not appear to interact directly, they share a number of proteins as common binding partners (Lee et al., 2018). Thus, we previously proposed that these three major post-synaptic proteins interact with each other to form a core scaffold, playing a crucial role in assembling dendritic spines at excitatory synapses (Lee et al., 2018).

**FIGURE 1:**
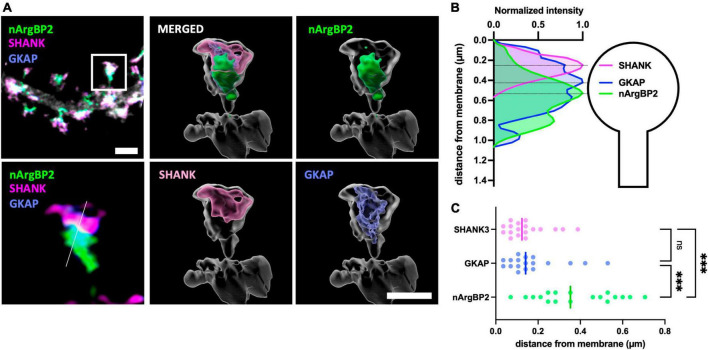
nArgBP2, GKAP and SHANK are spatially situated in distinct regions of the dendritic spines. **(A)** (Left) Super-resolution Airyscan images of dendritic spines labeled for nArgBP2 (green), SHANK (magenta) and GKAP (blue). Cultured hippocampal neurons were co-transfected with shRNA-nArgBP2, shRNA-resistant form of EGFP-nArgBP2, GKAP-Myc, HA-SHANK3, and followed by immunocytochemistry using specific antibodies against myc- and HA-tags. The lower panel is the enlarged image of an individual spine indicated with a rectangle in the upper panel. (Right) 3D volume-rendered images using Imaris. Scale bar: 2 and 1 μm. **(B)** Line scanning profile for each channel in an individual spine in **(A)**. **(C)** Quantification of the distance of each protein from the post-synaptic membrane (18 spines from 6 different neurons). Each shaded point represents the mean of the intensity profile from one spine and the lines in the middle represent the median. Friedman test followed by Dunn’s multiple comparisons test, p*** < 0.001.

To investigate their relative subcellular localizations within dendritic spines, we transfected primary cultured hippocampal neurons with shRNA-nArgBP2, shRNA-resistant form of EGFP-nArgBP2, GKAP-Myc, HA-SHANK3 and followed by immunocytochemistry using specific antibodies against Myc- and HA-tags. Subsequently, we captured images using Airyscan-based super-resolution microscopy (Figure 1). As expected, nArgBP2, GKAP, and SHANK3 were highly enriched in dendritic spines (Figure 1A). A previous study reported a high concentration of nArgBP2 at the PSD (Kawabe et al., 1999). However, we found that the majority of nArgBP2 localized in the lower stratum of dendritic spines, ∼ 500 nm from the surface, whereas SHANK3 was positioned closer to the membrane consistent with the previous studies (Grabrucker et al., 2014; Arons et al., 2016; Pfaender et al., 2017; Figure 1B). While GKAP appeared to have a broader distribution within spines compared to SHANK, the difference was not statistically significant (Figure 1C). We also found that a subset of nArgBP2 (∼ 20%) colocalized with SHANK3 in the upper stratum.

### nArgBP2, GKAP, and SHANK form distinctively localized spherical condensates in living fibroblasts

We recently showed that nArgBP2 undergoes phase separation *in vitro* and in living cells (Cho et al., 2023). PSD proteins including GKAP and SHANK also undergo LLPS *in vitro* (Zeng et al., 2018), and recent studies suggest that LLPS is a key mechanism for the subcellular organization of post-synaptic assembly (Chen et al., 2020). Given the distinct subcellular spatial arrangements we observed for nArgBP2, SHANK, and GKAP within dendritic spines (see Figure 1), we wondered how these proteins behave when co-expressed in living fibroblasts.

**FIGURE 2:**
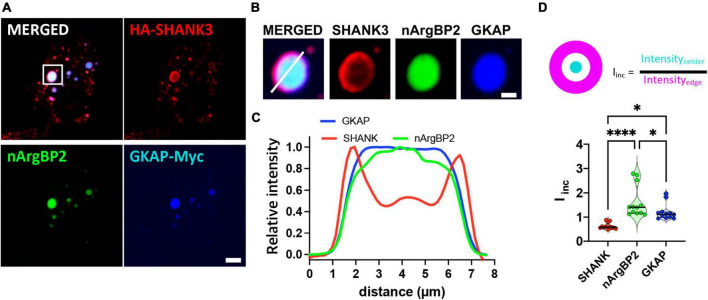
nArgBP2 along with SHANK3 and GKAP expressed in Cos7 cells form spherical condensates with distinct protein distribution patterns within the condensates. **(A)** Representative images of HA-SHANK3, EGFP-nArgBP2 and GKAP-Myc expressed in COS7 cells imaged 48 h after transfection. COS7 cells were co-transfected with EGFP-nArgBP2, HA-SHANK3, and GKAP-Myc, and subsequent immunostaining with specific antibodies against Myc- and HA-tags. Scale bar: 10 μm. **(B)** Enlarged images of droplet in panel **(A)**. Scale bar: 2 μm **(C)** Line scanning profile for each protein in panel **(B)**. **(D)** Incorporation index (I_inc_) for SHANK3, nArgBP2 and GKAP (See Methods for detail). HA-SHANK3 and GKAP-Myc were visualized by immunofluorescence staining. Data shown as violin plots, central bands represent the median and quartiles. *n* = 12. Friedman test followed by Dunn’s multiple comparisons test. *P*-values shown as *P < 0.05; ****P < 0.0001.

Co-transfection of COS7 cells with EGFP-nArgBP2, HA-SHANK3, and GKAP-Myc, and subsequent immunostaining with specific antibodies against Myc- and HA-tags revealed the formation of spherical condensates (Figure 2A). Strikingly, we further found distinct protein distribution patterns within the formed condensates (Figures 2B, C). Specifically, nArgBP2 concentrates predominantly in the inner phase of these condensates, while SHANK3 predominantly occupies the outer phase (Figure 2B). GKAP is distributed both in the outer phase and the inner phase (Figure 2B). Line scans and incorporation index conducted across the droplets documented the differential distribution profiles for each protein (Figures 2C, D), underscoring the resemblance between the observed sub-segregations within phase-separated droplets and the sub-cellular organization of these proteins in dendritic spines.

**FIGURE 3:**
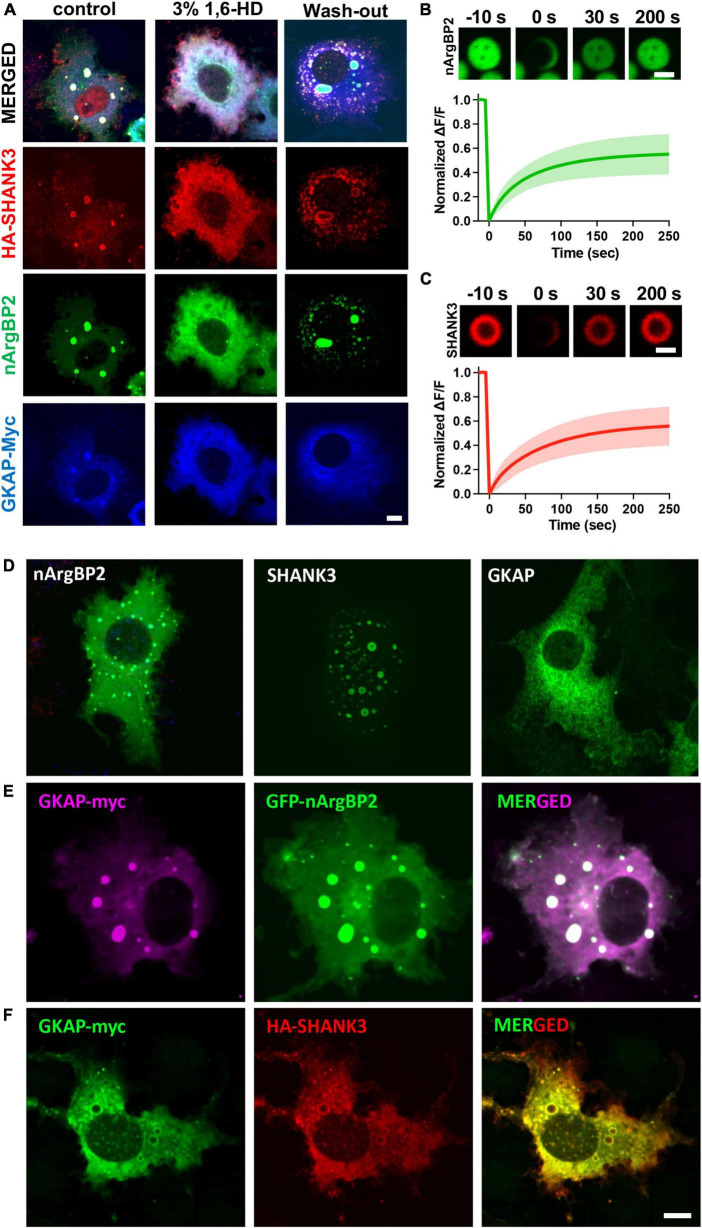
Liquid like behaviors of condensates and expression patterns nArgBP2, SHANK3 and GKAP when expressed alone or in conjunction with others. **(A)** Droplets dispersed upon treatment with 3% 1,6-Hexanediol (1,6-HD) and reformed after wash-out. Different cells were treated as indicated and fixed for the visualization of EGFP-nArgBP2, along with HA-SHANK3 and GKAP-Myc using immunofluorescence. **(B)** Representative time-lapse images showing fluorescence recovery after photobleaching EGFP-nArgBP2 and HA-SHANK3 droplets COS7 cells were co-transfected with EGFP-nArgBP2, HA-SHANK3, and mCherry-tagged frankenbody-HA (for live imaging of HA-SHANK3). FRAP was performed on different droplets due to variations in fluorescence intensity between EGFP-nArgBP2 and mCherry-frankenbody-labeled SHANK3. Scale bar: 2 μm. **(C)** Plots of normalized fluorescence intensity traces after photobleaching. Data are represented as mean ± SD (*n* = 8). **(D)** Representative images of EGFP-nArgBP2, HA-SHANK3 and GKAP-Myc, each singly expressed in Cos7 cells. **(E)** Co-expression pattern of GKAP-Myc and EGFP-nArgBP2 in Cos7 cell. **(F)** Co-expression pattern of GKAP-Myc and HA-SHANK3 in Cos7 cell. HA-SHANK3 and GKAP-Myc were visualized by immunofluorescence staining. Scale bar: 10 μm.

These condensates were dispersed by 3% 1,6-hexanediol (1,6-HD), an alcohol known to disperse various biomolecular condensates formed by LLPS through a mechanism involving its hydrophobicity (Lin et al., 2016; Kato and Mcknight, 2018; Figure 3A). Fluorescence recovery after photobleaching (FRAP) experiments also revealed that upon photobleaching, the EGFP-nArgBP2 or HA-SHANK3 (probed with co-transfected mCherry-tagged frankenbody-HA (Zhao et al., 2019) fluorescence recovered up to approximately 50% of the initial value (Figures 3B, C), indicating the liquid nature within condensates and dynamic exchange with the surrounding cytoplasm.

When SHANK3 is expressed alone, it also exhibits a propensity to form condensates but consistently positions itself in the periphery of these condensates (Figure 3D). This localization pattern persists even when co-expressed with either GKAP or nArgBP2. Conversely, GKAP, when expressed in isolation, does not form any discernible condensates (Figure 3D). However, the dynamics shift when co-expressed with either nArgBP2 or SHANK3, leading to the formation of condensates. Interestingly, the distribution of GKAP within these condensates is contingent upon its interaction partner. Specifically, it is evenly distributed in the presence of nArgBP2 (Figure 3E) or localized at the periphery in conjunction with SHANK3 (Figure 3F).

### CaMKII forms a distinct phase-in-phase droplet within SHANK3/GKAP or nArgBP2/SHANK3/GKAP droplets, which dissolves upon activation

CaMKIIα is a major effector enzyme in the PSD (Lisman et al., 2012; Hell, 2014), and has been implicated in intricate molecular interactions within biomolecular condensates formed by LLPS (Lee et al., 2009; Cai et al., 2021). CaMKIIα, when incorporated into the PSD-95-GluN2B-Stargazin condensate, induces the segregation of the Stargazin-PSD-95 protein condensate from the GluN2B-CaMKII protein condensate (Hosokawa et al., 2021). This segregation process ultimately gives rise to the formation of a nanodomain-like structure within a single protein condensate (Hosokawa et al., 2021). We also found that nArgBP2 was co-assembled into condensates with CaMKIIα in living COS7 cells and nArgBP2 condensates are regulated by CaMKIIα-mediated phosphorylation (Cho et al., 2023).

**FIGURE 4:**
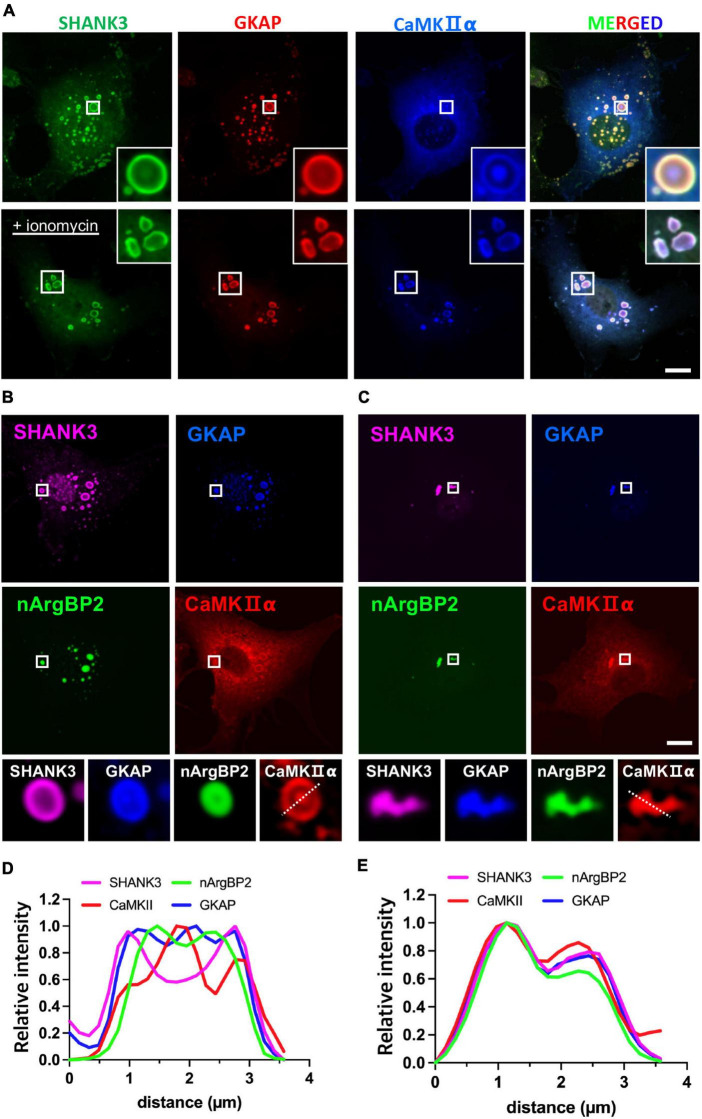
nArgBP2 with GKAP and SHANK3 forms dynamic assemblies regulated by CaMKIIα. **(A)** Representative images of HA-SHANK3, GKAP-Myc and CaMKIIα-SBFP2 co-expressed in COS7 cells. The lower panels show images after the treatment of ionomycin. The insets show enlarged images of rectangular regions. HA-SHANK3 and GKAP-Myc were visualized by immunofluorescence staining. Scale bar: 10 μm. **(B)** Representative images of HA-SHANK3, GKAP-Myc, EGFP-nArgBP2 and CaMKIIα-SBFP2 co-expressed in COS7 cells. The lower panels show enlarged droplets within rectangular regions. **(C)** Upon ionomycin treatment, the majority of droplets dispersed while the extant condensates underwent deformation into small irregular shapes, leading to the even distribution of all proteins within the condensates. HA-SHANK3 and GKAP-Myc were visualized by immunofluorescence staining. **(D,E)** Line scanning profiles for the enlarged droplets in the lower panels of panels **(B,C)**, respectively. Scale bar: 10 μm.

To test whether the inclusion of CaMKIIα may affect phase-separating behaviors of nArgBP2/SHANK/GKAP complex, we first expressed HA-SHANK3 and GKAP-Myc together with CaMKIIα-SBFP2 (Figure 4A). We found that SHANK3 continued to exhibit predominant localization in the outer region, and GKAP, which was localized along with nArgBP2 (Figures 2A–C), mostly resides in the outer region in the absence of ArgBP2 (Figure 4A). CaMKIIα, however, displayed a distinct localization pattern, positioning itself within the middle core, thus forming a discernible phase-in-phase organization. Notably, the regions between CaMKIIα and SHANK3/GKAP appeared to be devoid of any of these proteins. Upon activation of CaMKIIα by elevating cytosolic Ca^2+^ with ionomycin, the condensates underwent deformation but most notably, the phase-in-phase pattern of CaMKIIα in the central locus disappeared. Instead, CaMKIIα exhibited colocalization with SHANK and GKAP in the outer region (Figure 4A).

Upon additional co-expression of EGFP-nArgBP2, the localization patterns of SHANK3, GKAP, and CaMKIIα remained consistent with those observed in the absence of nArgBP2 (Figures 4B–E). nArgBP2 continued to exhibit enrichment within the inner phase of the condensates and CaMKIIα formed a phase-in-phase central droplet within the condensates. Additionally, mutual exclusivity between nArgBP2 and CaMKIIα was also observed, as CaMKIIα droplets appeared to be devoid of nArgBP2 (Figures 4B, D). Upon ionomycin treatment, the majority of droplets dispersed while the extant condensates underwent deformation into small irregular shapes, and sub-segregations observed within phase-separated droplets disappeared, leading to even distribution of all proteins within the condensates (Figures 4C, E). Together, these results suggest that nArgBP2, in conjunction with GKAP and SHANK3, autonomously organizes, leading to the formation of dynamic layered assemblies, and that this process is regulated by CaMKIIα.

## Discussion

Here, we show that nArgBP2, GKAP, and SHANK3 exhibit distinctive localization within the dendritic spine in a layered distribution. Specifically, nArgBP2 is situated in the lower stratum of dendritic spines near the spine core, while SHANK3 and GKAP are positioned close to the post-synaptic membrane. We further show that nArgBP2, in conjunction with SHANK3 and GKAP self-organizes and assembles biomolecular condensates in COS7 cells. nArgBP2 was exclusively enriched in the inner phase of the condensates while SHANK3 and GKAP were primarily located in the outer area. We further found that CaMKIIα underwent phase-separation with them but forms a distinct phase-in-phase central condensate, which subsequently disappeared upon ionomycin treatment.

The PSD, characterized in electron micrographs as an approximately 30 nm thick electron-dense structure just beneath the post-synaptic membrane (Chen et al., 2008), additionally encompasses a deeper layer referred to as the “pallium,” housing a scaffold of SHANK and Homer proteins. Our data indicate that the majority of nArgBP2 is situated at approximately 300–600 nm from the post-synaptic membrane, particularly within the spine core. However, biochemical analysis in a previous study revealed its enrichment in the PSD fraction (Kawabe et al., 1999). Potential factors contributing to the observed discrepancy may include: (1) variations between cultured neurons and *in vivo* observations, (2) nArgBP2 binding to GKAP, or (3) contamination of the isolated PSD fraction with deeper spine cytosol components. However, we also cannot rule out the possibility that the exogenous expression of nArgBP2 may lead to a broader expression pattern than the endogenous one. We also found that GKAP seems to be broadly present in dendritic spines compared to SHANK although statistically not significant (Figure 1C). A previous EM study reported contrasting findings, with GKAP distributed near the post-synaptic membrane and SHANK having a wider distribution extending deeper into the cytoplasm (Tao-Cheng et al., 2015). This inconsistency may stem from incomplete fixation and/or antibody staining in the dense PSD fraction or differences in overexpression levels between HA- and Myc-tagged proteins. The spine core is a specialized compartment responsible for maintaining structural integrity and stability. This aligns with the major function of nArgBP2, which regulates actin cytoskeletons for spine formation during development and remodeling during synaptic plasticity (Lee et al., 2016; Cho et al., 2023). Additionally, we observed a subset of nArgBP2 co-localized with SHANK3 in the upper stratum (Figure 1), indicating the strategic localization of nArgBP2 in both upper and lower strata of spines, underscoring its dual functionality as a scaffold linker and actin-regulating protein within dendritic structures.

Our study reveals an interesting finding: CaMKIIα forms distinct phase-in-phase droplets within condensates composed of SHANK3/GKAP or SHANK3/GKAP/nArgBP2 (Figure 4). This contrasts with previous *in vitro* studies where purified SHANK3 and CaMKIIα, when co-incubated, formed even-distributed condensates and were subsequently recruited into NR2B and PSD-95 condensates upon stimulation (Cai et al., 2021). Notably, CaMKIIα was observed translocating to condensate peripheries when calcium decreased (Hosokawa et al., 2021), suggesting its ability to shuttle between two PSD subcompartments in response to Ca^2+^. Our experiments showed unique phase-in-phase CaMKIIα droplets within SHANK3/GKAP or SHANK3/GKAP/nArgBP2 condensates (Figure 4), and notably, co-expression with nArgBP2 filled the previously protein-free region between CaMKIIα and SHANK3/GKAP. Our findings differ from prior *in vitro* work, likely due to the complexity introduced by numerous additional proteins that may fill the observed gap in the intervening region. Despite differences, our findings suggest CaMKIIα’s potential to shuttle between distinct PSD subcompartments in response to stimulation. Further investigation is needed to explore interactions among various PSD proteins and CaMKII within the context of phase separation.

We hypothesized that nArgBP2, GKAP, and SHANK constitute a core scaffolding triad orchestrating multiple protein interactions in dendritic spines. Supporting this, studies suggest pivotal roles for these proteins in synaptic function (Naisbitt et al., 1999; Shin et al., 2012; Lee et al., 2016). These proteins may establish a dynamic molecular framework, acting as a molecular hub to coordinate various intracellular signaling pathways and influence overall synaptic strength. The intricate interplay among nArgBP2, GKAP, and SHANK significantly contributes to the structural and functional plasticity of synapses, underscoring their importance in maintaining a finely tuned excitatory synaptic network.

In conclusion, we provide evidence that nArgBP2 exhibits spatial localization distinct from SHANK3 and GKAP within the dendritic spines. The relative localizations of these proteins in spines are readily evident in the autonomously established layered organizations within condensates in living fibroblasts. Although the understanding of how the phase-separating behaviors of post-synaptic proteins precisely dictate the layered post-synaptic organization in neurons is still limited, our results, combined with previous findings, propose a strong functional correlation between these phenomena. This certainly requires further investigation, particularly in the context of their functional implications in synaptic plasticity and neurological disorders.

## Data availability statement

The original contributions presented in this study are included in this article/supplementary material, further inquiries can be directed to the corresponding authors.

## Ethics statement

The animal study was approved by the Institute of Animal Care and Use Committee (IACUC, Approval ID number: SNU-100930-5) of Seoul National University, Korea. The study was conducted in accordance with the local legislation and institutional requirements.

## Author contributions

S-EL: Conceptualization, Data curation, Formal analysis, Investigation, Methodology, Validation, Writing−original draft. SC: Conceptualization, Funding acquisition, Supervision, Validation, Writing−original draft.
